# The BRCA1 Ubiquitin ligase function sets a new trend for remodelling in DNA repair

**DOI:** 10.1080/19491034.2016.1267092

**Published:** 2016-12-29

**Authors:** Ruth M. Densham, Joanna R. Morris

**Affiliations:** Birmingham Centre for Genome Biology and Institute of Cancer and Genomic Sciences, Medical and Dental School, University of Birmingham, Edgbaston, Birmingham, United Kingdom

**Keywords:** BRCA1, homologous recombination, resection, SMARCAD1, ubiquitin

## Abstract

The protein product of the breast and ovarian cancer gene, BRCA1, is part of an obligate heterodimer with BARD1. Together these RING bearing proteins act as an E3 ubiquitin ligase. Several functions have been attributed to BRCA1 that contribute to genome integrity but which of these, if any, require this enzymatic function was unclear. Here we review recent studies clarifying the role of BRCA1 E3 ubiquitin ligase in DNA repair. Perhaps the most surprising finding is the narrow range of BRCA1 functions this activity relates to. Remarkably ligase activity promotes chromatin remodelling and 53BP1 positioning through the remodeller SMARCAD1, but the activity is dispensable for the cellular survival in response to cisplatin or replication stressing agents. Implications for therapy response and tumor susceptibility are discussed.

## Introduction

The BRCA1 protein plays several roles in genome stability: including check-point promotion, DNA cross-link repair, replication fork stability and DNA double-strand break (DSB) repair. In DSB repair it is associated with homologous recombination (HR). Here it promotes DNA resection by two means: interacting with the resection protein CtIP; and by opposing the block on resection contributed by the p53 binding protein 53BP1 and its effectors (reviewed in^1,2^). In addition it aids RAD51 loading through interaction with PALB2-BRCA2.^3,4,5^ In the absence of BRCA1, DSBs are repaired by toxic non-homologous end joining (NHEJ).^6^

The first 100 amino acids of BRCA1 encodes a RING domain (Really Interesting New Gene) and lengthy α helices. The latter form a hydrophobic bundle with the similarly arranged N-terminal region of BARD1^7^ while the RING interacts with E2 Ub conjugating enzymes and promotes the transfer of ubiquitin (Ub) from the E2 to a target protein.^8,9^ Here we review recent insights into BRCA1-BARD1 function as an E3 Ub ligase and the role this activity is thought to play in contributing to DNA repair.

### How do RING E3 Ubiquitin ligases work?

**Figure 1.**
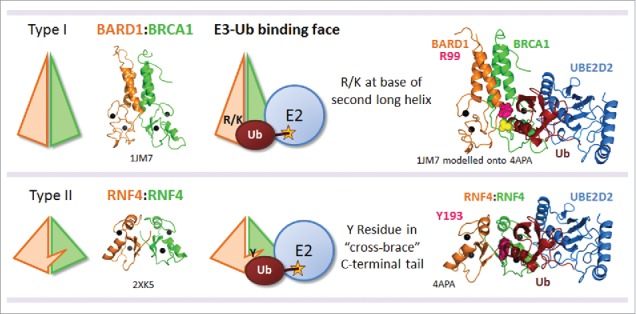
The Ubiquitin binding face on dimeric RING E3 ligases. There are two major types of dimeric RING E3 ligases. Type I dimerize by interactions with extended α-helical tails, illustrated here by BRCA1-BARD1. Type II exhibit an interleaved “cross brace” structure, illustrated here by RNF4. In both cases the second non-E2 bound RING provides an additional Ub binding interface that helps to “lock” the E2∼Ub complex into a closed conformation, and thereby promoting Ub transfer. (PDB: 1JM7,^7^ 4PPE^64^ and 4AP4^11^).

Unlike the HECT family of E3 Ub ligases those of the RING family do not form a catalytic intermediate with Ub. Historically, RING E3 Ub ligases were considered passive players in Ub conjugation, providing enzyme stability, substrate specificity to the E2 conjugating enzyme but having no active role in Ub transfer. However, foundational work from several laboratories revealed that RING-bearing Ub ligases contribute by priming a loaded E2∼Ub complex for Ub transfer.^10-15^ Dimeric RING E3 Ubiquitin ligases can be split into two structural classes^16^: those with an interleaved “cross-brace” C-terminal tail sometimes referred to as Type II (eg RNF4 and BIRC7); and those with extended α-helical tails important for dimerization, referred to as Type I (eg BRCA1-BARD1, the Polycomb Repressor Complex 1(PRC1) Ligase RING1A/B-BMI1/MEL-18 and Rad18). In Type II dimeric RINGs an additional E3-Ub interface lies within conserved residues in the “cross-brace” tail from the second non-E2 bound RING protomer^11,14^ (Fig. 1). This direct interaction restrains free movement of Ub in the E2∼Ub complex, thereby “locking” Ub into a closed conformation primed to stimulate Ub transfer. However since Type I dimeric RINGs lack a “cross-brace” feature, any additional Ub binding face from the second RING must necessarily differ.

Recently, the Ub-E3 binding face in Type I RING E3 ligases was identified.^17,18^ In these structures a conserved positive residue (R/K) at the base of the second long α-helix is required for ligase activity. Strikingly, this conserved residue is missing in the E2 bound partner of the BRCA1-BARD1 and PRC1 complexes but present in their inactive binding partners (eg. BARD1 and BMI1) in an arrangement similar to that described for Type II active structures. Therefore, the need to form a closed E3-E2∼Ub complex via interaction with the conserved Ub-binding face may explain the requirement of heterodimerisation for enzymatic activity in these complexes. Indeed, in the case of the PRC1 ligase complex, activity correlates with the accessibility of the conserved R/K residue.^18^ In BMI1, the conserved residue K73 forms a salt bridge with neighboring D77 correlating with auto-inhibited low ligase activity. Mutation of K73 to Arginine disrupts this internal salt bridge and increases ligase activity. By selecting partners with either a K or R at this conserved site, RING1A/B can switch activity states from low to high. More broadly, mutation of this E3-Ub binding interface in dimeric RINGs can be applied to selectively control ligase activity and interrogate enzyme function in the context of a stable dimer. We recently utilized this approach to explore the role of BRCA1-BARD1 ligase activity in the DNA damage response.^17^

### BRCA1-ligase activity acts to counter chromatin barriers

BRCA1 has several roles in DNA repair (reviewed in^19-21^), reflected in the sensitivities of cells lacking BRCA1 to a broad range of DNA damaging agents. It was intriguing to discover that only a subset of these agents require BRCA1-BARD1 ligase activity for full resistance. Indeed, cells expressing a stable heterodimer that lacks ligase activity showed sensitivity to olaparib, camptothecin, etoposide and IR, but resistance to replication stressing agents, hydroxyurea and aphidicolin, and resistance to ICL agent, cisplatin. Intriguingly chicken DT40 cells expressing a form of BRCA1 defective for interaction with E2 conjugating enzymes also showed camptothecin and etoposide sensitivity, but was resistant to the ICL agent mitomycin-C.^22^

Ligase-defective cells showed reduced, but not eliminated, RPA and RAD51 foci in S-phase cells after IR. Consistent with a defect in these foci signifying reduced ssDNA and Rad51-filament formation respectively, we found that the lengths of BrdU labeled ssDNA, used as a direct measure of resection lengths, were shortened in cells expressing ligase defective BRCA1-BARD1. In normal S-phase cells, BRCA1 acts to counter the 53BP1-mediated block on resection, thereby promoting HR.^6,23,24^ Since ligase defective cells showed reduced resection in S-phase it was perhaps unsurprising to find that co-depletion of 53BP1 circumvented the need for BRCA1-BARD1 ligase activity in olaparib and camptothecin resistance, RAD51 foci formation and in full length resection.

Previous reports have shown that at S-phase damage foci, BRCA1 forms an internal “core” and is required to direct 53BP1 to the foci periphery.^25,26^ This remodelling of 53BP1 correlates with the appearance of RPA in the foci core which is thought to indicate permissive resection. Intriguingly, in cells complemented with ligase defective BRCA1-BARD1, 53BP1 no longer occupies the periphery but instead co-locates with BRCA1 at the core suggesting that E3 Ub ligase activity, rather than BRCA1 occupation itself, is associated with 53BP1 repositioning.

Together these data place a role for BRCA1s ligase activity at the heart of the BRCA1–53BP1 antagonistic relationship to control resection - a key component in determining HR pathway choice.

### H2A as a BRCA1 ligase target is functionally related to resection

**Figure 2.**
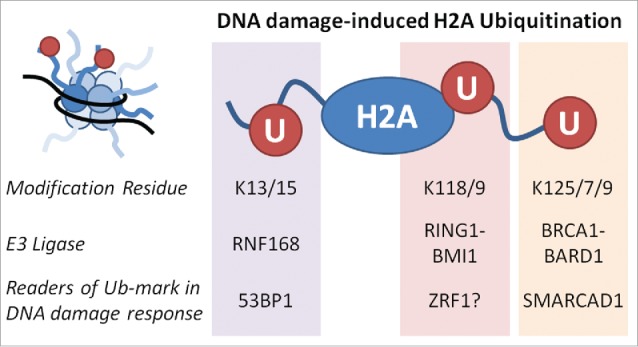
DNA damage-induced H2A ubiquitination. H2A has three major sites of ubiquitination which targeted by specific E3 ligases^29,38,65^ and readers^17,31,66,67^ in the DNA damage response.

The targets of BRCA1 E3 ligase activity identified to date include histones (H2A, H2AX) RNA polII, TFIIE, NPM1, CtIP, gamma-tubulin, ER-α and claspin (reviewed in^27^). Histone ubiquitination in the DNA damage directs the ordered recruitment of repair proteins to damage sites^28^ (Fig. 2). RNF168 is responsible for H2A K13/15 modification^29,30^ that contributes to 53BP1 interaction with nucleosomes around sites of broken DNA.^31^ PRC1 is responsible for much of the H2A ubiquitination in cells, but is also actively recruited to sites of DNA damage^32^ through PRC2^33^ and CBX4^34^ where the K118/119 modification is associated with local transcriptional repression,^33,35^ and may also promote the ubiquitin signaling pathway that subsequently recruits BRCA1 and 53BP1.^32^ Several groups have shown H2A is a BRCA1 target,^36-38^ which our work confirmed.^17^ BRCA1-dependent conjugation sites have been mapped to the extreme C-terminus of H2A at K125/127/129.^38^ Thus in the context of DNA damage BRCA1 contributes the third Ub modification on this histone in relation to the DDR.

Since the BRCA1-BARD1 Ub-modification site on H2A lies at its extreme C-terminus, we and others^37^ have made use of H2A-Ub C-terminal fusion proteins to try and rescue the phenotypes of BRCA1-BARD1 deficient cells. The laboratory of Inder Verma showed that expression of such a fusion restored growth defects and a measure of gene-conversion in BRCA1-deficient cells.^37^ We extended these observations and found a C-terminal H2A-Ub fusion, but not an N-terminal fusion, was able to restore RAD51 foci formation and both Olaparib and camptothecin resistance in cells lacking BRCA1-BARD1 suggesting that H2A is a major target of BRCA1-BARD1 ligase activity.

### H2A-Ub link to chromatin remodelling

H2A ubiquitination events contribute to transcriptional repression and act as a platform for protein complex formation. Since BRCA1-BARD1 ligase activity promotes 53BP1 remodelling to the periphery of damage foci, we considered the possibility that chromatin remodellers mediate this process.

**Figure 3.**
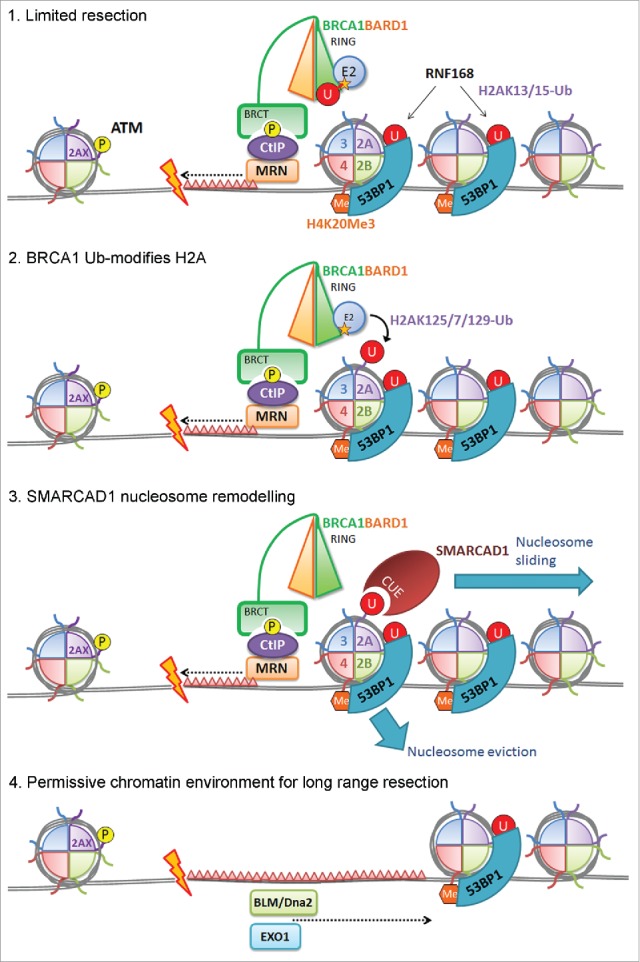
Proposed contribution of the BRCA1 ligase activity to steps in DNA resection. Limited resection occurs in the absence of BRCA1-BARD1 activity dependent on CtIP-Mre11. BRCA1-BARD1 dependent Ub modification of H2A promotes SMARCAD1 interaction with damage-proximal nucleosomes. SMARCAD1 activity repositions or evicts nucleosomes moving 53BP1 and its effector proteins to release 53BP1-mediated inhibition of DNA resection. Long range resection can proceed.

The human homolog of the yeast SWI/SNF-like chromatin remodeller Fun30, SMARCAD1, has been implicated in long-range resection.^39^ In yeast the need for Fun30 is lessened if the resection-block provided by the 53BP1 ortholog, rad9, is removed.^40^ We were intrigued by the presence of two N-terminal ubiquitin-binding CUE domains in SMARCAD1^41,42^ and examined whether SMARCAD1 links BRCA1-BARD1 ligase function and H2A modification to 53BP1 positioning and resection. SMARDCA1 recruitment to laser-induced DNA damage was partially dependent on both BRCA1-BARD1 and the CUE domains, and depletion of SMARCAD1 was epistatic with BRCA1-BARD1 loss showing reduced resection. Further, SMARCAD1 promotes 53BP1 repositioning to foci periphery in a manner that requires both the ATPase and CUE domains. Together our data place SMARCAD1 in a cascade down-stream of BRCA1-BARD1 histone ubiquitination and up-stream of the promotion of 53BP1 positioning and long-range resection (Fig. 3).

### Not a pure reader of H2A-Ub

SMARCAD1 co-purifies nucleosomes in which H2A carries a C-terminal Ub fusion from cell extracts. However, further investigation of SMARCAD1 *in vitro* using recombinant core nucleosomes revealed no increased binding affinity for Ub-modified H2A compared with unmodified-H2A (pers. comm. Michael Ucklemann & Titia Sixma, NKI Ntherlands) as previously noted.^43^ This may indicate requirements for additional nucleosome contacts (for example, linker H1^44^), bridging between nucleosomes, or added specificity from another histone modification event missing *in vitro*.

### Chromatin in resection

The ATPase activity of SMARCAD1 is required for 53BP1 positioning and Olaparib resistance,^17^ but whether this is due to nucleosome sliding or eviction of H2A:H2B from nucleosomes is unclear. Fun30 is able to promote both activities^43^ and either would be expected to result in apparent repositioning of 53BP1. Nucleosomes are themselves inhibitory to long range resection *in vitro*,^45^ although removal of 53BP1 and its associated factors, is sufficient to allow resection in BRCA1-deficient or BRCA1-ligase defective cells, suggesting they do not pose a significant block alone. SMARCAD1 co-purifies with several other remodelling factors^46^ associated with gene silencing and heterochromatin formation, some of which have also been implicated in 53BP1 repositioning,^47^ perhaps implying the existence of a larger complex or network. An emerging theme in 53BP1 repositioning is an association with factors previously implicated in transcriptional silencing.

### What's the limit?

Recently Ochs et al.^48^ suggested that 53BP1, in marking a limit to resection, both controls resection lengths for HR and prevents excessive resection that leads to RAD52-mediated single strand annealing and chromosome re-arrangements.^48^ Thus the question of how the spread of 53BP1 positioning is restricted arises. One answer may be the limited spread of BRCA1 at sites of damage which may geographically bound resection. Another would be an opposing deubiquitination of the C-terminal H2A-Ub mark. Multiple DUBs have been implicated in the removal of ubiquitin from H2A (reviewed in^49^) whether one or several of these contribute to resection inhibition remains to be seen. Further, histone exchange may also play a role. The incorporation of H2AZ at sites of damage has been proposed to limit resection and define chromatin boundaries.^50^ Interestingly, H2AZ, like H2AX, lacks the BRCA1-BARD1 K125/7/9 H2A Ub sites and therefore, may provide a resection boundary that is naturally refractory to SMARCAD1 remodelling – an idea that remains to be tested.

We understand comparatively little about the relative positioning of many of the factors critical to the regulation of resection including the 53BP1-binding proteins responsible for the block on resection. It would be intriguing to establish, for example, where RNF168 and K63-Ub chains are in relation to peripheral 53BP1 and whether histone relationships are altered prior to, or in conjunction with, resection.

### Chromatin context and the requirement for ligase activity

Ahead of the replication fork two nucleosomes are normally destabilized.^51^ In the context of processing DSBs that occur as a consequence of replication fork collision with ssDNA breaks or protein-DNA complexes, formed as a result of Topoisomerase or PARP poisoning, further chromatin remodelling is clearly required. Further, cells treated with PARP inhibitor may be more dependent on the BRCA1-BARD1 pathway to recruit chromatin remodellers, since PARylation is required for the recruitment of the chromatin remodeller ALC1 to sites of DNA damage.^52^

Importantly while BRCA1 promotes ICL repair, replication fork stability and restart, these processes appear BRCA1-BARD1 ligase independent.^17^ Further, neither expression of a H2A-Ub fusion nor co-depletion of 53BP1 was able to rescue heterodimer-deficient cell survival sensitivities to these agents,^17,53^ implying that a different chromatin context and alternative BRCA1-dependent pathways are required for repair. Sensitivity to replication stalling agents, such as hydroxyurea, occurs following a programmed break generated by structure-specific endonucleases likely to be active at a regressed fork in which nucleosomes are assembled.^54^ Survival is mediated by HR-mediated replication restart and new origin firing. ICL repair is replication-dependent and occurs once two replication forks converge, presumably producing an initial structure of a pair of regressed, chromatinized forks.^55^ After the lesion is unhooked, translesion synthesis repairs one strand and the other is repaired through HR.^19^

Currently we can only speculate why these contexts do not require ligase activity. Perhaps the same level of resection is not needed, or other factors direct remodelling, or, alternatively, the underlying chromatin state is sufficiently open to be permissive for resection. Intriguingly FANCJ can counteract chromatin compaction associated with replication.^56^ Further it is clear that SMARCAD1 is present behind the replication fork and interacts with PCNA^46^ where it may not need BRCA1, or the H2A-Ub mark for its localization and activity. Many other chromatin remodellers and histone chaperones are also active in this context. Finally, DNA topology may contribute to a refractory or permissive state. The front of the fork is positively supercoiled and factors such as the 53BP1 complex may be more able to restrict nuclease access, whereas behind the fork negatively supercoiled DNA may lessen their repressive impact.

### Links to epigenetic gene silencing?

Following replication SMARCAD1 acts to promote deacetylation of newly synthesized histones in the restoration of heterochromatin.^46^ Its depletion reduces markers of heterochromatin at satellite repeats.^44^ SMARCAD1 interacts with PCNA,^46^ but there is also a contribution of the CUE-domain to localization, which may or may not relate to PCNA.^44^ It is intriguing to note BRCA1 loss similarly impacts heterochromatin at satellite repeats and that expression of an H2A-Ub fusion is able to restore satellite DNA silencing.^37^ For this reason it would be interesting to know whether chronic SMARCAD1 recruitment and activity in replication contexts is influenced by BRCA1, and whether this contributes to the altered heterochromatin state reported in BRCA1 deficient cells.^37^

### RING-Less BRCA1

Recent reports of RING-less forms of BRCA1, expressed in cells using internal, downstream ATG sites^57-59^ are intriguing in view of the role of the BRCA1 E3 Ub ligase function, encoded by the RING and by BARD1 interaction. These RING-less proteins cannot interact with BARD1 yet are stable due to deletion of the degron within the heterodimer interface while expression of BARD1 itself is lost. We might predict that these proteins would retain many of the functions of full length BRCA1 but can have no ligase activity and would be expected to lack the ability to counter 53BP1 and promote long-range resection.

Mice lacking BRCA1 exon two die in early embryogenesis despite expressing a stable RING-less BRCA1, produced from a down-stream ATG at Methionine 90/99. Their lethality is rescued on a 53BP1−/− background indicating murine embryos without the RING die due to the presence of 53BP1. Comparison of cells bearing a conditional deletion of exon2 on a WT or 53BP1−/− background after Olaparib treatment showed excessive chromosome aberrations when 53BP1 was present.^58^ In a second murine model, tumors expressing RING-less BRCA1 (on a 53BP1 WT background) exhibited levels of RPA recruitment to sites of damage at levels similar to a full BRCA1 knockout indicating resection remains impaired.^57^ Moreover mice homozygous both for the exon 2 loss and 53BP1 loss exhibited no tumor susceptibility.^58^ These observations are consistent with a critical RING-encoded function of BRCA1 directing opposition to 53BP1, thereby promoting resection competence and preventing genome instability, embryonic lethality and possibly tumor growth.

The founder mutation BRCA1–185delAG results in a highly truncated protein but selection of tumors and cells with DNA damaging agents does not induce secondary mutations in BRCA1 or 53BP1 but instead promotes a switch to expression of a RING-less BRCA1 initiated from Methionine 90/99 in mouse and 279 in humans.^57,59^ A human cell line bearing this mutation SUMO1315, expresses a RING-less form of BRCA1 and is cisplatin resistant^57^ and can be made more so by further overexpression of the RING-less BRCA1.^59^ The majority of tumors from mice bearing BRCA1-del185AG also rapidly became cisplatin resistant. These observations are consistent with the ICL–repair function of BRCA1 residing outside the RING portion of BRCA1.

These studies have also provided a considerable surprise. Despite RING-less BRCA1 tumors exhibiting defective RPA, RAD51 foci are present, albeit at severely reduced intensities.^57^ Similarly, B cells conditionally deleted for BRCA1 exon 2 exhibit RAD51 foci and their levels of sister chromatin exchanges following olaparib treatment are comparable to WT cells, suggesting functional HR.^58^ In human cells the promotion of RAD51 foci by RING-less BRCA1 is also evident.^57,59^ This restoration of HR has implications and murine tumors expressing RING-less BRCA1 show only a partial response to olaparib compared with those lacking full length BRCA1. In cell models comparing PARP inhibitor sensitivities, RING-less contributes a fold less resistance then full length BRCA1, but 30x more than cells without re-expression. In xenografts expression of RING-less BRCA1 results in a failure to respond to PARP inhibitor treatment^59^ and in PDX models BRCA1-del185AG bearing tumors show a poor response to olaparib.^60^

Whether resection remains defective in all these RING-less models remains to be seen, but they indicate that expression, and overexpression often seen in adaptation,^59^ of a protein retaining the majority of BRCA1 functions (such as CtIP interaction and PALB2-binding), is sufficient to promote some HR. We speculate that it is possible this occurs by utilizing shorter ssDNA lengths. Alternatively a greater proportion of lesions may be dealt with in a chromatin environment where the ligase activity is not needed, perhaps by awaiting replication fork convergence in late S-phase. Both strategies would be likely to be error-prone.

### Role of ligase activity in tumor susceptibility?

Finally, whether the ligase activity of BRCA1-BARD1 alone is required to prevent tumor development remains an open question. The data from animals lacking exon 2, but expressing RING-less BRCA1^58^ suggest chromosome aberrations may accumulate if 53BP1 is not lost, thereby providing a mechanism for tumor development. They argue that resolved HR provided by RING-less BRCA1 is insufficient to restore embryonic viability. Chronic loss of ligase activity may also alter transcriptional repression, contributing to tumor development.^37^ However the BRCA1 murine model, BRCA1-I26A, in which BRCA1 interaction with E2 Ub conjugating enzymes is reduced, is viable and not tumor prone,^61,62^ and if the fidelity of HR promoted by other regions of BRCA1 remains high this may explain why tumor development is stymied. Supporting this perspective SMARCAD1 knockout mice have several defects, but are viable.^63^ Definitive evidence for or against cancer protection denoted by BRCA1-BARD1 Ub ligase activity awaits further investigation.
